# Externally validated explainable 3D CNN ensemble model for non-invasive prediction of IDH and MGMT status in gliomas

**DOI:** 10.3389/fonc.2026.1790776

**Published:** 2026-08-26

**Authors:** Rafail C. Christodoulou, Georgios Vamvouras, Rafael Pitsillos, Kleoniki Roka, Elena E. Solomou, Christina Kalogeropoulou, Peter Zampakis, Michalis F. Georgiou

**Affiliations:** 1 Department of Radiology, Stanford University School of Medicine, Stanford, CA, United States; 2 Department of Mechanical Engineering, National Technical University of Athens, Zografou, Greece; 3 Department of Bioinformatics, Cyprus Institute of Neurology and Genetics, Nicosia, Cyprus; 4 Department of Pediatric Oncology, University of Athens Medical School, Athens, Greece; 5 Department of Internal Medicine-Hematology, University of Patras Medical School, Rion, Greece; 6 Department of Radiology, University of Patras Medical School, General Hospital of Patras, Patras, Greece; 7 Department of Radiology, Division of Nuclear Medicine, University of Miami, Miami, FL, United States

**Keywords:** convolutional neural network, gliomas, IDH, magnetic resonance imaging, MGMT

## Abstract

**Introduction:**

To develop and externally validate an explainable 3D convolutional neural network (CNN) ensemble for non-invasive prediction of IDH mutation and MGMT promoter methylation status in gliomas using routine MRI.

**Methods:**

Multi-institutional MRI data were curated from the UCSF-PDGM and UPENN-GBM cohorts for training and testing, with independent external validation on the MU-Glioma-Post dataset. Separate 3D CNNs were trained for IDH and MGMT classification, and the models were combined into an ensemble metaclassifier. Model interpretability was assessed using Integrated Gradients saliency maps for voxel-level attributions and Shapley values for modality-level contributions.

**Results:**

The ensemble model achieved an AUC of 0.94 (95% CI, 0.84–1.00) in testing and 0.77 (95% CI, 0.71–0.82) in external validation for IDH mutation classification. The ensemble yielded AUCs of 0.81 (95% CI, 0.64–0.94), 0.68 (95% CI, 0.47–0.88), and 0.57 (95% CI, 0.52–0.63) for MGMT promoter methylation in validation, testing, and external validation, respectively. Integrated Gradients (IG) maps highlighted biologically plausible regions, with IDH predictions focusing on peritumoral FLAIR hyperintensity and MGMT predictions emphasizing the enhancing tumor core on T1CE. Shapley analysis showed FLAIR contributed 64% and T1CE 36% to IDH classification, while MGMT classification relied more heavily on T1CE (60.6%), with complementary input from FLAIR (30.8%).

**Discussion:**

We present an explainable 3D ensemble deep learning framework capable of predicting IDH mutation status from routine MRI with moderate external generalizability. While the same framework achieved strong internal performance for MGMT methylation status, this did not generalize on external validation, underscoring that MGMT prediction from imaging alone remains an unresolved challenge requiring further methodological development.

## Graphical Abstract

**Figure:**
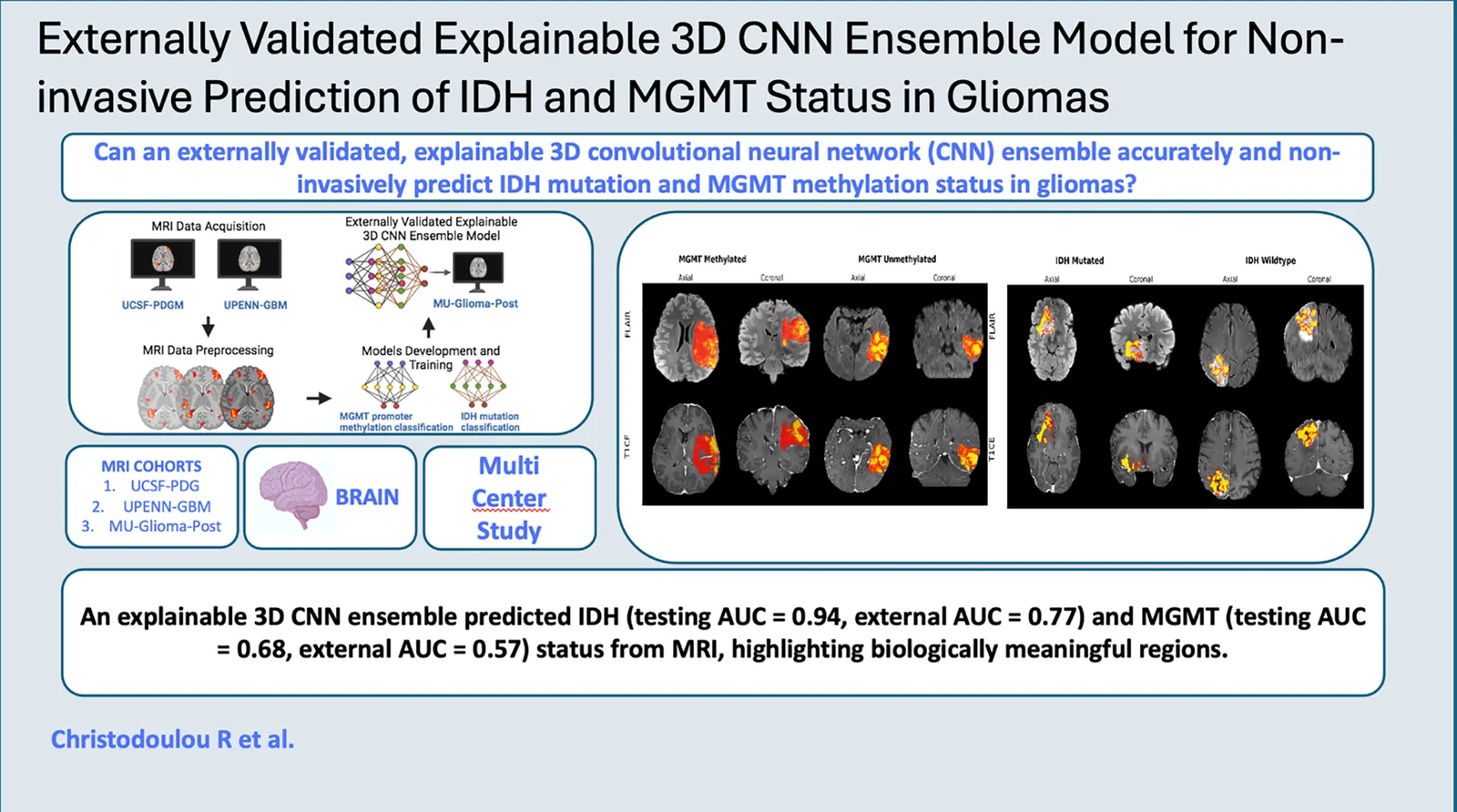

## Introduction

Gliomas are the most common primary CNS tumors, arising from glial cells such as astrocytes and oligodendrocytes. According to CBTRUS, from 2017 to 2021, the incidence of CNS primary tumors was 25 cases per 100,000, with gliomas making up about 23% (1). The accumulation of genetic and molecular changes in glial cells leads to their neoplastic transformation and infiltration into brain tissue, resulting in characteristic histological features.

The latest WHO (5th edition) highlights molecular testing in glioma subtypes, with IDH mutation status being key for classifying diffuse glioma groups (2). IDH mutation and MGMT methylation assessment have significant prognostic and predictive value in tumor progression. MGMT encodes O6-methylguanine-DNA methyltransferase, a DNA repair enzyme that removes cytotoxic alkyl adducts from the O6 position of guanine, counteracting the effect of alkylating chemotherapy. Epigenetic silencing of the MGMT promoter by methylation reduces expression of this repair enzyme, impairing the tumor’s capacity to reverse alkylation-induced DNA damage and thereby sensitizing cells to agents such as temozolomide (3). Detecting IDH mutations is crucial for therapeutic decision-making, as selective IDH inhibitors are now in clinical use.

Despite the clinical importance of molecular biomarkers, their detection often requires invasive tissue sampling, which can limit their use in specific clinical settings. Tumor heterogeneity complicates the diagnostic process, as intratumoral regions exhibit considerable variation in their molecular and genetic profiles (4). These challenges highlight the growing need for non-invasive neuroimaging-based approaches to predict pre-operative glioma molecular profiles based on IDH mutation and MGMT gene promoter methylation.

The value of neuroimaging in tumor detection is well established. Core protocols, as defined by the Brain Tumor Imaging Protocol, introduced in 2015, include 3D T1-weighted pre- and post-contrast, T2-weighted, and FLAIR sequences (5, 6). These MRI sequences reveal macroscopic signs of molecular pathology, providing insights into tumor biology.

Building on this foundation, deep learning (DL) techniques have emerged, automatically learning hierarchical, non-linear feature representations from raw data (7), enabling effective molecular profile prediction despite intratumoral heterogeneity. CNN models were subsequently utilized. Most studies employing 2D CNNs or smaller architectures do not capture the complex spatial understanding of the tumor environment. This limitation led to the use of 3D CNN models to predict tumor molecular features. Several studies investigated these techniques, offering robust, high-accuracy models (8–10).

This study introduces a 3D deep learning model that predicts glioma biomarkers, such as IDH mutation and MGMT methylation, from MRI data. It assesses both markers simultaneously, using a single pipeline, and captures the entire tumor better than 2D models. The model was trained, validated, and tested on the preoperative UCSF-PDGM and UPenn-GBM cohorts and externally evaluated on the post-treatment MU-Glioma-Post cohort, which tests robustness under treatment-related domain shift. IG heat maps enhance interpretability for clinical use, enabling accurate, non-invasive tumor profiling for pre-operative and treatment planning.

## Materials and methods

### Image acquisition

MRI data for training, validation, and testing were obtained from the UCSF-PDGM and UPENN-GBM (11, 12) cohorts, while the MU-Glioma-Post dataset (13) was used for external validation. UCSF-PDGM and UPENN-GBM comprise preoperative examinations, whereas MU-Glioma-Post consists exclusively of post-treatment MRI acquired after surgical resection and, in most cases, adjuvant chemoradiation; it contains no treatment-naive baseline studies and provides a resection cavity segmentation label in addition to enhancing tumour, non-enhancing necrotic core, and non-enhancing FLAIR signal hyperintensity (14). External validation was therefore performed under a deliberate treatment-related domain shift rather than as a preoperative replication cohort. Scanner hardware and acquisition parameters are detailed in the **Supplementary Material** under the ‘Scanner and Acquisition Parameters’ section.

### Preprocessing

Data were pre-processed in four steps. First, volumes were cropped around the tumor using the segmentation mask. Second, MGMT samples included segmentation, T1CE, and FLAIR as three input channels, whereas IDH samples included only T1CE and FLAIR. In both tasks, T1CE and FLAIR were multiplied by the segmentation mask to remove non-tumor tissue (15). Thus, segmentation was required for preprocessing and prospective inference. UPENN masks were manual when available and automated otherwise. The remaining steps included percentile-based normalization and training augmentation. In MGMT, methylated was class 1 and unmethylated was class 0; in IDH, mutated was class 1 and wildtype was class 0. More preprocessing details are in the **Supplementary Material** under “*Detailed preprocessing steps*”.

### Descriptive statistics

The unified UCSF and UPENN datasets were split into training, validation, and testing sets, as shown in **Supplementary Table 1**. Similarly, descriptive statistics are provided in **Table 1** for the MGMT and IDH cases, for the training, validation, testing, and external validation source datasets. The difference in sample number between cases is due to IDH and MGMT status not being jointly available for all samples, as in many cases, only one of the two was reported. Therefore, the IDH and MGMT models were trained and evaluated on biomarker-specific cohorts rather than identical patient subsets. Tumor samples were analyzed for IDH mutations through genetic sequencing of biopsy or resection material. At the same time, MGMT promoter methylation was determined in grade III and IV gliomas using a methylation-sensitive quantitative PCR method.

**Table 1:** Population statistics for the source datasets, reported at the unique-case level.

MGMT status classification source datasets						
Characteristic	UCSF (n=312)		UPENN (n=232)		MU (n=482)	
Age Mean ± SD	59.4 ± 13.6		62.7 ± 12.3		57.1 ± 15.3	
Male	200	64.1%	142	61.2%	279	57.9%
Female	112	35.9%	90	38.8%	203	42.1%
MGMT unmethylated	113	36.2%	152	65.5%	295	61.2%
MGMT methylated	199	63.8%	80	34.5%	187	38.8%
MGMT indeterminate	0	0.0%	0	0.0%	0	0.0%
IDH wildtype	62	19.9%	59	25.4%	398	82.6%
IDH mutated	29	9.3%	6	2.6%	71	14.7%
IDH indeterminate	221	70.8%	167	72.0%	13	2.7%
IDH status classification source datasets						
Characteristic	UCSF (n=198)		UPENN (n=226)		MU (n=558)	
Age Mean ± SD	50.0 ± 16.4		62.0 ± 13.4		56.8 ± 15.0	
Male	125	63.1%	144	63.7%	322	57.7%
Female	73	36.9%	82	36.3%	236	42.3%
MGMT unmethylated	29	14.6%	39	17.3%	288	51.6%
MGMT methylated	62	31.3%	26	11.5%	181	32.4%
MGMT indeterminate	107	54.0%	161	71.2%	89	15.9%
IDH wildtype	95	48.0%	114	50.4%	462	82.8%
IDH mutated	103	52.0%	112	49.6%	96	17.2%
IDH indeterminate	0	0.0%	0	0.0%	0	0.0%

Cases with indeterminate or unknown biomarker status were excluded from model training, validation, testing, and external evaluation for that biomarker. These cases were retained only for descriptive reporting in **Table 1**. Because IDH and MGMT labels were not always jointly available, a case could be included for one biomarker while excluded for the other.

### Explainability

Voxel-wise importance heatmaps for the FLAIR and T1CE modalities were created using Integrated Gradients to demonstrate each voxel’s significance to the classification output. More details are available under the “*Explainability*” section of the **Supplementary Material**.

Channel-wise contributions were computed using exact Shapley values, reported as percentages per sample and averaged across the test set to capture the underlying trend. MGMT Shapley values were computed across segmentation, T1CE, and FLAIR channels; IDH Shapley values were computed only across T1CE and FLAIR.

### External validation

In each case, the selected models and the ensemble metaclassifiers were evaluated on data from the MU-Glioma-Post dataset, which was pre-processed in the same way as the training, validation, and testing data. They were assessed in the same leakage-proof manner as the test set, using the already computed, validation-optimized thresholds. Because MU-Glioma-Post contains no preoperative examinations, the external evaluation could not be restricted to treatment-naive imaging, and post-surgical cavities, blood products, and post-radiation signal change were present in the externally evaluated volumes.

ROC AUC uncertainty was estimated using stratified nonparametric bootstrap resampling with 10,000 replicates within each validation, testing, and external validation set, preserving class counts. The 95% confidence interval was defined by the 2.5th and 97.5th percentiles of the bootstrap distribution. To assess whether each ensemble improved discrimination beyond the best individual model, ensemble AUC was compared with the highest-AUC single model within the same split using paired bootstrap resampling of the same cases; differences are reported as ensemble AUC minus best single-model AUC. Given class imbalance, especially in the external IDH cohort, ensemble performance was also assessed using precision-recall curves, average precision, calibration plots, and Brier scores. Calibration curves were interpreted descriptively because of the small internal validation and testing sets.

### Part A: MGMT promoter methylation classification

#### Model development and training

Binary classification used a 3D CNN in TensorFlow Keras. Despite differences in architectural and training hyperparameters, the core structure was consistent. The input first passed through a convolutional feature extractor with Conv3D and MaxPooling3D layers. The output is then connected to either a GlobalAveragePooling3D or a Flatten layer. Flatten preserves spatial layout but increases the number of parameters and the risk of overfitting, while GAP creates a compact, lower-parameter representation. These outputs fed into fully connected layers with variable activation and dropout, culminating in a Dense layer with a single neuron and a Sigmoid activation for binary classification. Training loss was binary cross-entropy, and the optimizer was ADAM with a variable learning rate, reduced during training via the ReduceLROnPlateau callback. To control memory usage, data were provided to the network via a custom Keras Sequence data generator that optionally loaded each mini-batch from disk when needed and released the memory after use. The batch size was set to 1 due to hardware constraints, and early stopping was applied by monitoring the Area Under the Curve (AUC) on the validation set. The threshold of each individual MGMT model was selected after training by maximizing class-1 F1 score on the validation set. The selected threshold was then fixed and applied unchanged to the testing and external validation sets.

#### Hyperparameter optimization

Model architecture and training-specific hyperparameters were optimized using Optuna (16). More details are available under the “*Hyperparameter Optimization for Convolutional MGMT status Classifiers*” section and **Supplementary Tables 2, S3**.

#### Ensemble metaclassifier

After 500 Optuna trials for individual MGMT model development, four high-validation-AUC models were chosen to create an ensemble metaclassifier based on XGBClassifier, following scaling with StandardScaler. The metaclassifier takes individual class-1 probabilities and produces a unified estimate, aggregating information from multiple models for better accuracy. More details are available under the “*Hyperparameter Optimization for MGMT Ensemble Metaclassifier*” section of the **Supplementary Material**.

### Part B: IDH mutation classification

#### Model development and training

**Figure 1:**
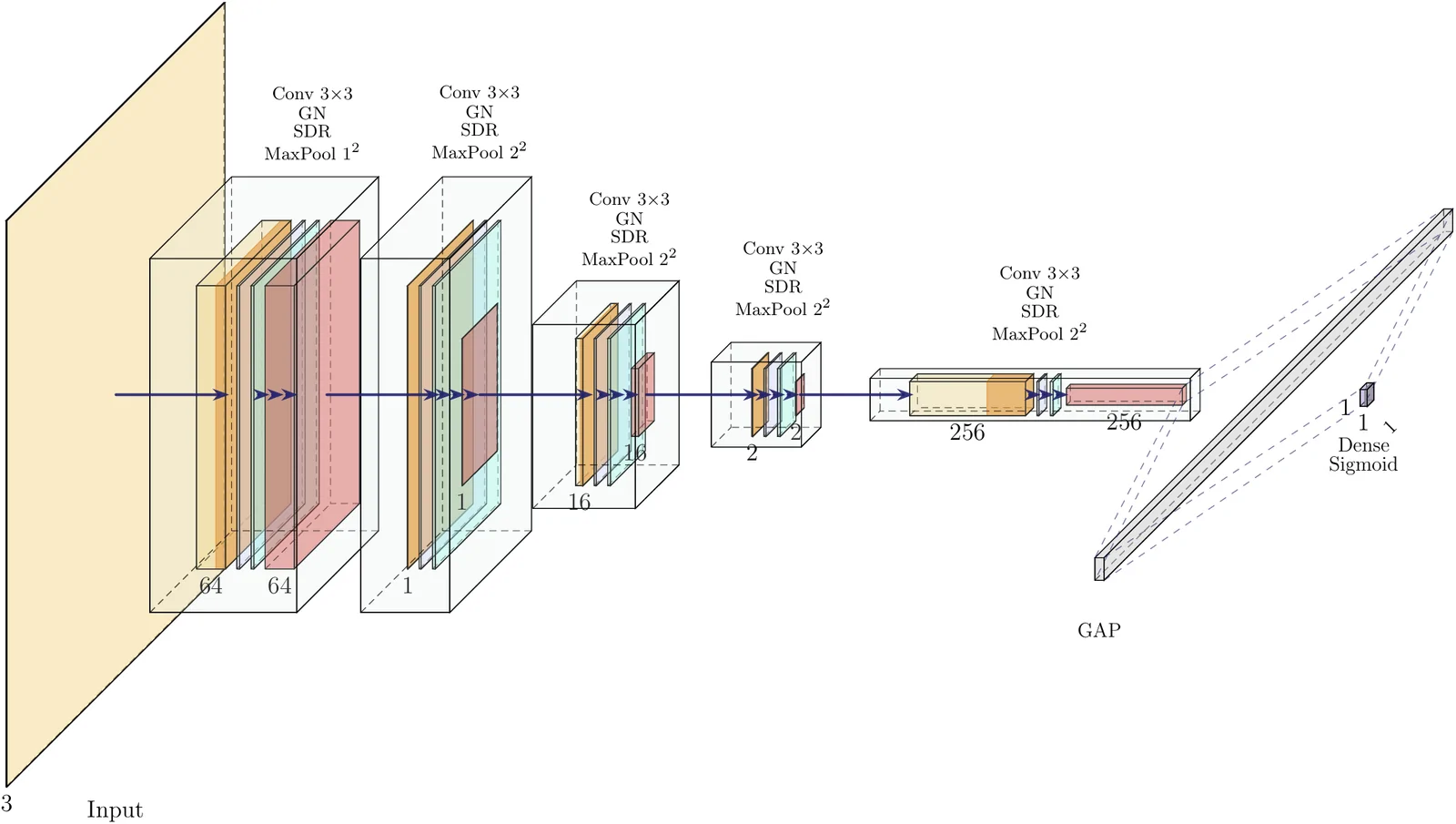
Architecture of the best-performing individual model in IDH mutation status classification. The network consists of five convolutional blocks with Group Normalization and ReLU activations, incorporating SpatialDropout3D and L2 kernel regularization across layers. The convolutional blocks use filter sizes of 64, 1, 16, 2, and 256 with 3×3 kernels and progressive max pooling, resulting in an overall 16× spatial reduction. The convolutional feature maps are aggregated through Global Average Pooling and connected directly to a single sigmoid neuron for binary classification.

The model structure differed from MGMT despite identical training settings. After input, a configurable number of convolutional blocks processed data, each with a Conv3D (with optional L2 regularization), group normalization, activation, spatial dropout, and downsampling via max pooling or a strided Conv3D. Only one block used the second downsampling method, determined during hyperparameter tuning. Spatial dropout was added to deactivate a percentage of channels to increase robustness. Following the blocks, a GlobalAveragePooling3D layer, configurable Dense layers, and one final Dense layer produced class-1 probability (**Figure 1**). Thresholds for individual IDH models were selected on validation data by minimizing the squared sum of the false-positive rate and false-negative rate, then fixed and applied unchanged to the testing and external validation sets.

#### Hyperparameter optimization

Model architecture and training-specific hyperparameters were optimized using Optuna. More details are available under the “*Hyperparameter Optimization for Convolutional IDH Status Classifiers*” section and **Supplementary Tables 6, S7**.

#### Ensemble metaclassifier

After 500 Optuna trials for IDH model development, 13 high-validation-AUC architectures were selected to create a weighted ensemble metaclassifier. The IDH ensemble threshold was selected on the validation set using Youden’s J and was then fixed for testing and external validation. This metaclassifier received the individual model probabilities as input and outputted an improved probability equal to an optimally weighted average of the inputs. More details are available under the “*IDH Ensemble Metaclassifier*” section of the **Supplementary Material**.

## Results

### Part A: MGMT promoter methylation classification

#### Classification

**Figure 2:**
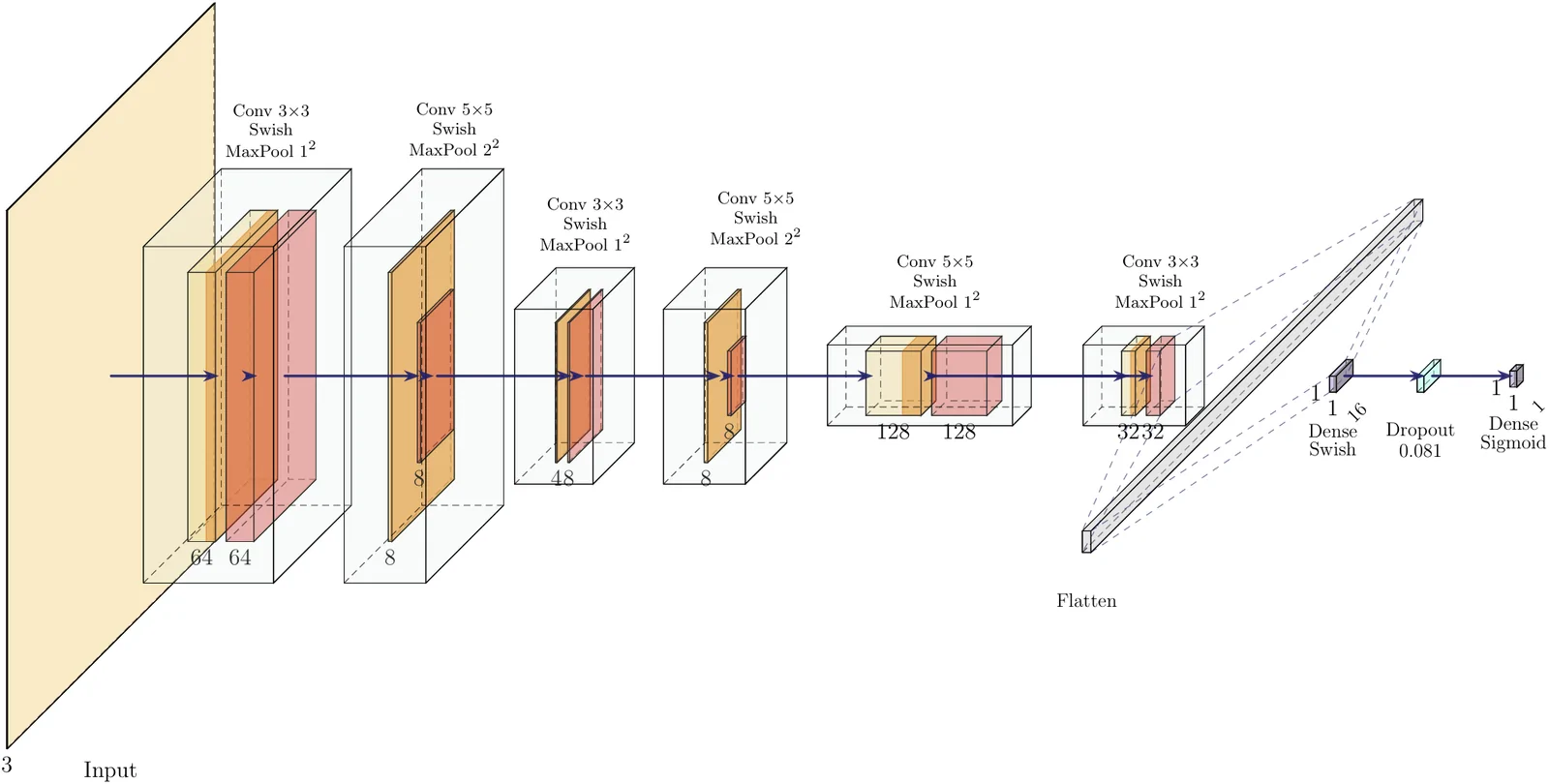
Architecture of model 2, the highest-performing model in MGMT methylation status classification. The model consists of six convolutional blocks with max pooling (active or inactive), and a flatten-type head connecting the convolutional section to the fully connected layers, followed by a dense layer of 16 units with Swish activation, dropout of 0.081, and a final sigmoid neuron for binary classification.

**Figure 3:**
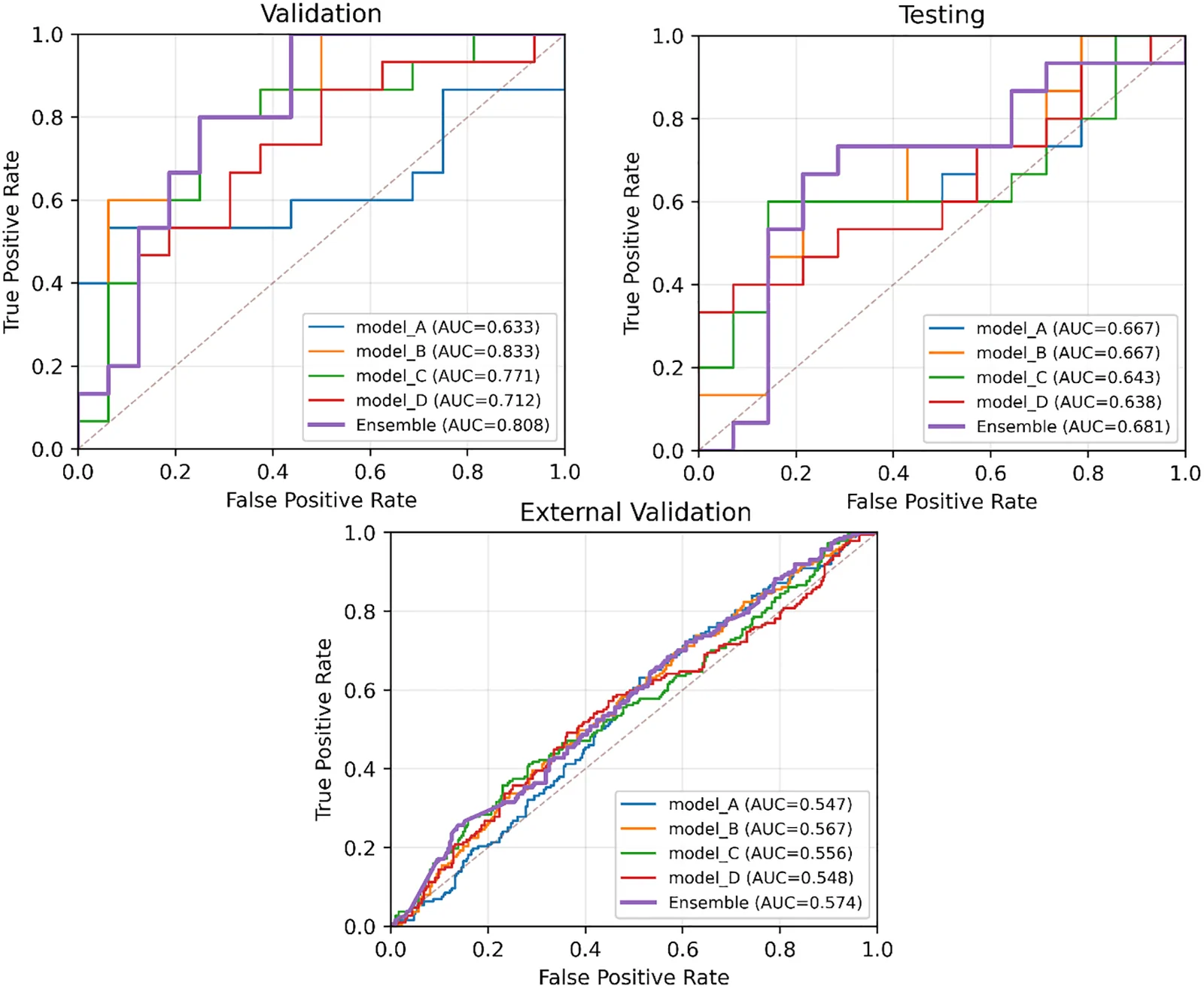
ROC AUC curves for each set for individual models and the ensemble metaclassifier.

**Table 2** presents metrics for four models selected by validation AUC, with **Figure 2** showing the architecture of Model 2, which had the best individual validation performance. **Figure 3** displays ROC AUC curves for each model and the ensemble. AUCs are reported with 95% confidence intervals in **Table 2**. The ensemble did not clearly outperform the best individual MGMT model. Relative to the best single model, the ensemble AUC differences were -0.025 (95% CI, -0.117 to 0.050) in validation, 0.014 (95% CI, -0.219 to 0.252) in testing, and 0.007 (95% CI, -0.013 to 0.028) in external validation. We therefore interpret the ensemble as a model-combination summary rather than evidence of superior MGMT discrimination. The ensemble metaclassifier’s validation AUC was 0.81 (95% CI, 0.64-0.94), testing AUC was 0.68 (95% CI, 0.47-0.88), and external validation AUC was 0.57 (95% CI, 0.52-0.63). The decline in external AUC is expected due to domain, population, and label shifts. Precision-recall and calibration analyses are shown in **Supplementary Figures 1** and **2**, with summary metrics in **Supplementary Table 8**. The MGMT ensemble average precision was 0.769, 0.655, and 0.450 in validation, testing, and external validation, respectively. Corresponding Brier scores were 0.264, 0.291, and 0.403, supporting limited external probability reliability for MGMT.

**Table 2:** MGMT classification results.

Dataset	Metric	Model 1	Model 2	Model 3	Model 4	Ensemble
	Threshold	0.400	0.529	0.600	0.471	0.830
Validation	ROC AUC (95% CI)	0.63 (0.41-0.83)	0.83 (0.68-0.96)	0.77 (0.58-0.93)	0.71 (0.52-0.89)	0.81 (0.64-0.94)
	Confusion Matrix	$\left[\begin{array}{cc} 0 & 16\\ 0 & 15 \end{array}\right]$	$\left[\begin{array}{cc} 8 & 8\\ 0 & 15 \end{array}\right]$	$\left[\begin{array}{cc} 12 & 4\\ 3 & 12 \end{array}\right]$	$\left[\begin{array}{cc} 4 & 12\\ 1 & 14 \end{array}\right]$	$\left[\begin{array}{cc} 9 & 7\\ 0 & 15 \end{array}\right]$
	F1	0.65	0.79	0.77	0.68	0.81
	Precision	0.48	0.65	0.75	0.53	0.68
	Recall	1.00	1.00	0.80	0.93	1.00
Testing	ROC AUC (95% CI)	0.67 (0.45-0.85)	0.67 (0.46-0.85)	0.64 (0.42-0.84)	0.64 (0.42-0.83)	0.68 (0.47-0.89)
	Confusion Matrix	$\left[\begin{array}{cc} 0 & 14\\ 0 & 15 \end{array}\right]$	$\left[\begin{array}{cc} 7 & 7\\ 4 & 11 \end{array}\right]$	$\left[\begin{array}{cc} 10 & 4\\ 6 & 9 \end{array}\right]$	$\left[\begin{array}{cc} 3 & 11\\ 3 & 12 \end{array}\right]$	$\left[\begin{array}{cc} 10 & 4\\ 4 & 11 \end{array}\right]$
	F1	0.68	0.67	0.64	0.63	0.73
	Precision	0.52	0.61	0.69	0.52	0.73
	Recall	1.00	0.73	0.60	0.80	0.73
External	ROC AUC (95% CI)	0.55 (0.50-0.60)	0.57 (0.51-0.62)	0.56 (0.50-0.61)	0.55 (0.50-0.60)	0.57 (0.52-0.63)
	Confusion Matrix	$\left[\begin{array}{cc} 0 & 295\\ 0 & 187 \end{array}\right]$	$\left[\begin{array}{cc} 132 & 163\\ 68 & 119 \end{array}\right]$	$\left[\begin{array}{cc} 164 & 131\\ 90 & 97 \end{array}\right]$	$\left[\begin{array}{cc} 36 & 259\\ 26 & 161 \end{array}\right]$	$\left[\begin{array}{cc} 164 & 131\\ 87 & 100 \end{array}\right]$
	F1	0.56	0.51	0.47	0.53	0.47
	Precision	0.39	0.42	0.43	0.38	0.53
	Recall	1.00	0.64	0.52	0.81	0.43

#### Explainability

**Figure 4:**
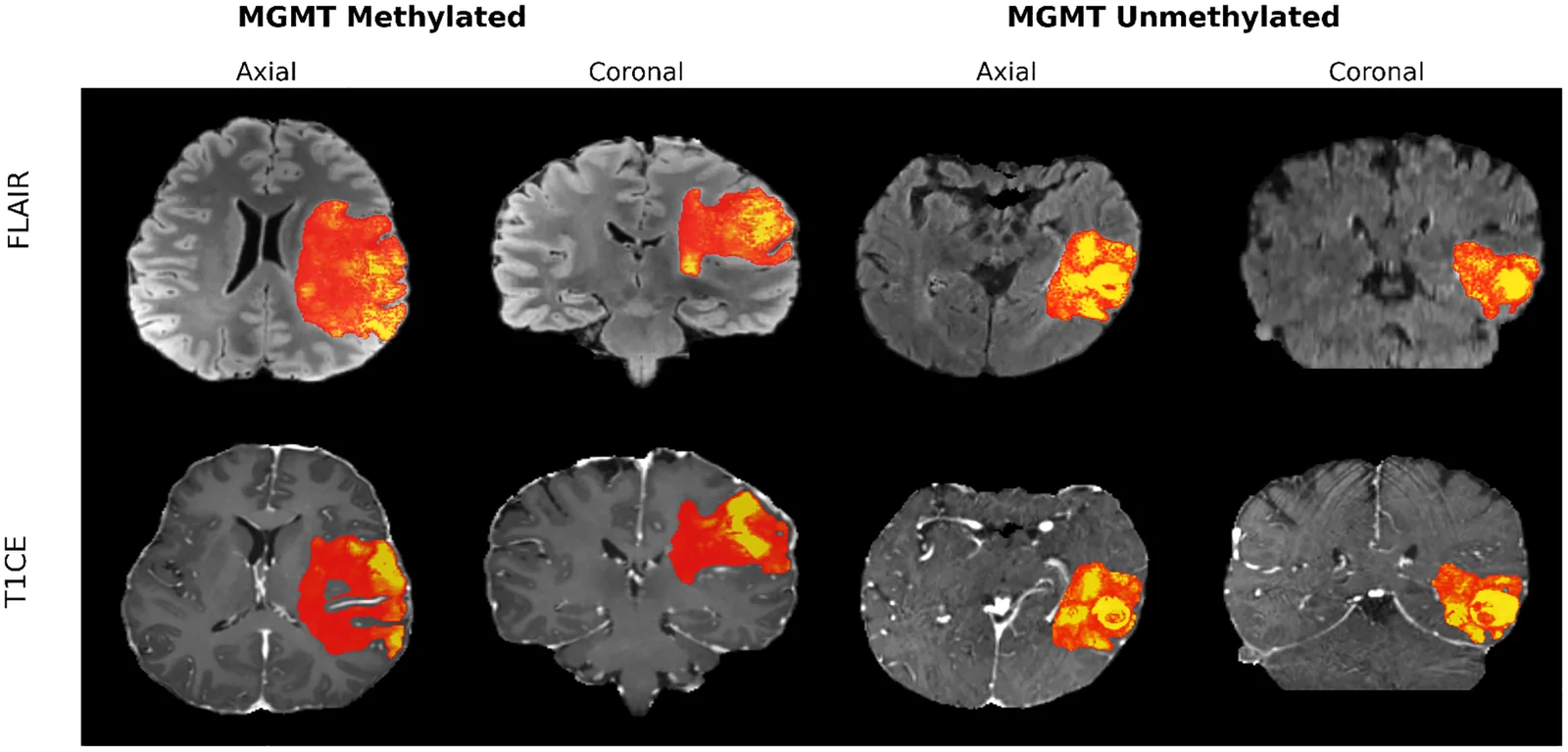
Representative Integrated Gradient saliency maps for MGMT promoter methylation prediction in two patients. Axial and coronal slices of FLAIR (top row) and contrast-enhanced T1-weighted (T1CE, bottom row) MR images are displayed for MGMT-methylated (left) and unmethylated (right) gliomas. Warm colors (yellow–red overlays) indicate voxels with higher attribution to the model’s prediction.

The IG analysis results are shown in **Figure 4** for one representative MGMT-methylated and one unmethylated case across FLAIR and T1CE sequences. High-intensity attributions (yellow) were concentrated within the tumor area, particularly in the enhancing core on T1CE and along the hyperintense tumor-peritumoral interface on FLAIR. Although MGMT status does not directly determine imaging appearance, unmethylated gliomas are typically more aggressive and often present with greater peritumoral edema and heterogeneous enhancement compared with methylated tumors (17, 18). This trend was reflected in the IG maps, where a larger proportion of voxels within FLAIR and T1CE contributed to the model’s prediction in unmethylated cases.

The computation of exact Shapley values quantified the relative contribution of each MRI modality to the classification outcome, yielding that the segmentation channel contributed minimally (8.6%). In comparison, T1CE (60.6%) and FLAIR (30.8%) accounted for most of the predictive weight. This cohort-level modality analysis indicates that the model relied most heavily on T1CE, with complementary information from FLAIR. The IG maps provide representative spatial examples of these modality-level findings.

### Part B: IDH mutation classification

#### Classification

**Figure 5:**
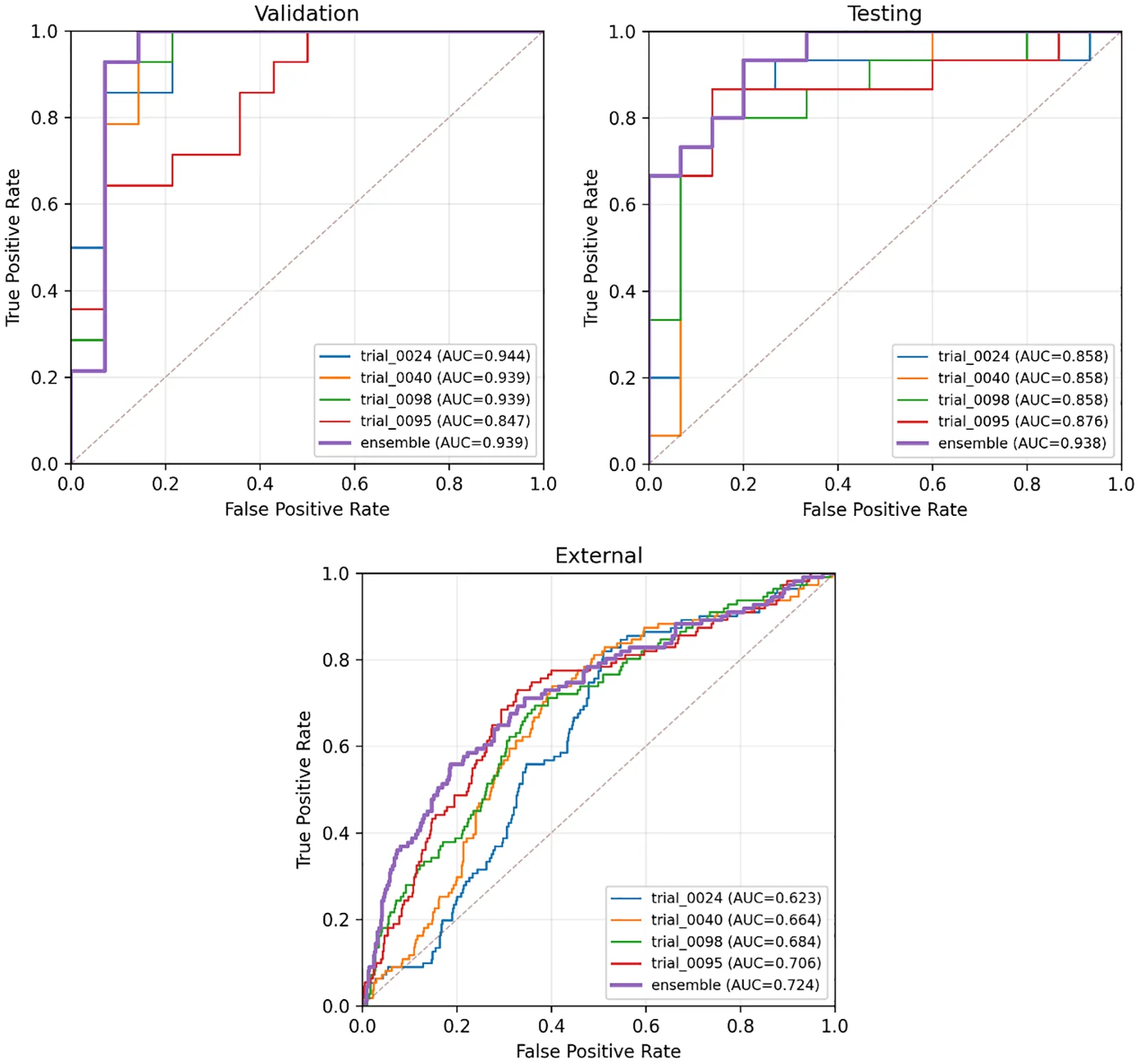
ROC AUC curves for each set for individual models and the ensemble metaclassifier.

The metrics are shown in **Table 3**, with AUCs reported with 95% confidence intervals. ROC AUC curves for models and the ensemble are in **Figure 5** across validation, testing, and external sets. Among the individual models, Model 1 had the highest validation AUC, while Model 4 had the highest testing and external validation AUCs. The ensemble metaclassifier had a validation AUC of 0.94 (95% CI, 0.81-1.00), a testing AUC of 0.94 (95% CI, 0.84-1.00), and an external validation AUC of 0.77 (95% CI, 0.71-0.82). The ensemble did not demonstrate statistically clear superiority over the best individual model in paired bootstrap comparisons: AUC differences were -0.005 (95% CI, -0.077 to 0.061), 0.062 (95% CI, -0.027 to 0.187), and 0.030 (95% CI, -0.007 to 0.067) in validation, testing, and external validation, respectively. Thus, the ensemble results should be interpreted cautiously at these sample sizes. The external IDH set was imbalanced, with 462 wildtype and 96 mutated cases. The IDH ensemble average precision was 0.908, 0.944, and 0.418 in validation, testing, and external validation, respectively. Corresponding Brier scores were 0.191, 0.174, and 0.235. These findings support good internal discrimination but more cautious interpretation of external probabilities.

**Table 3:** IDH classification results.

Dataset	Metric	Model 1	Model 2	Model 3	Model 4	Ensemble
	Threshold	0.469	0.425	0.488	0.444	0.424
Validation	ROC AUC (95% CI)	0.94 (0.84-1.00)	0.94 (0.83-1.00)	0.94 (0.82-1.00)	0.85 (0.68-0.96)	0.94 (0.81-1.00)
	Confusion Matrix	$\left[\begin{array}{cc} 13 & 1\\ 2 & 12 \end{array}\right]$	$\left[\begin{array}{cc} 12 & 2\\ 0 & 14 \end{array}\right]$	$\left[\begin{array}{cc} 13 & 1\\ 1 & 13 \end{array}\right]$	$\left[\begin{array}{cc} 11 & 3\\ 4 & 10 \end{array}\right]$	$\left[\begin{array}{cc} 13 & 1\\ 1 & 13 \end{array}\right]$
	F1	0.889	0.933	0.929	0.741	0.929
	Precision	0.923	0.875	0.929	0.769	0.929
	Recall	0.857	1.000	0.929	0.714	0.929
Testing	ROC AUC (95% CI)	0.86 (0.69-0.99)	0.86 (0.69-0.99)	0.86 (0.70-0.98)	0.88 (0.71-0.99)	0.94 (0.84-1.00)
	Confusion Matrix	$\left[\begin{array}{cc} 12 & 3\\ 3 & 12 \end{array}\right]$	$\left[\begin{array}{cc} 13 & 2\\ 2 & 13 \end{array}\right]$	$\left[\begin{array}{cc} 13 & 2\\ 3 & 12 \end{array}\right]$	$\left[\begin{array}{cc} 12 & 3\\ 2 & 13 \end{array}\right]$	$\left[\begin{array}{cc} 13 & 2\\ 3 & 12 \end{array}\right]$
	F1	0.800	0.867	0.828	0.839	0.828
	Precision	0.800	0.867	0.857	0.812	0.857
	Recall	0.800	0.867	0.800	0.867	0.800
External	ROC AUC (95% CI)	0.65 (0.60-0.70)	0.69 (0.65-0.74)	0.72 (0.67-0.78)	0.74 (0.68-0.79)	0.77 (0.71-0.82)
	Confusion Matrix	$\left[\begin{array}{cc} 148 & 314\\ 6 & 90 \end{array}\right]$	$\left[\begin{array}{cc} 158 & 304\\ 4 & 92 \end{array}\right]$	$\left[\begin{array}{cc} 178 & 284\\ 10 & 86 \end{array}\right]$	$\left[\begin{array}{cc} 156 & 306\\ 11 & 85 \end{array}\right]$	$\left[\begin{array}{cc} 163 & 299\\ 10 & 86 \end{array}\right]$
	F1	0.360	0.374	0.369	0.349	0.358
	Precision	0.223	0.232	0.232	0.217	0.223
	Recall	0.938	0.958	0.896	0.885	0.896

#### Explainability

**Figure 6:**
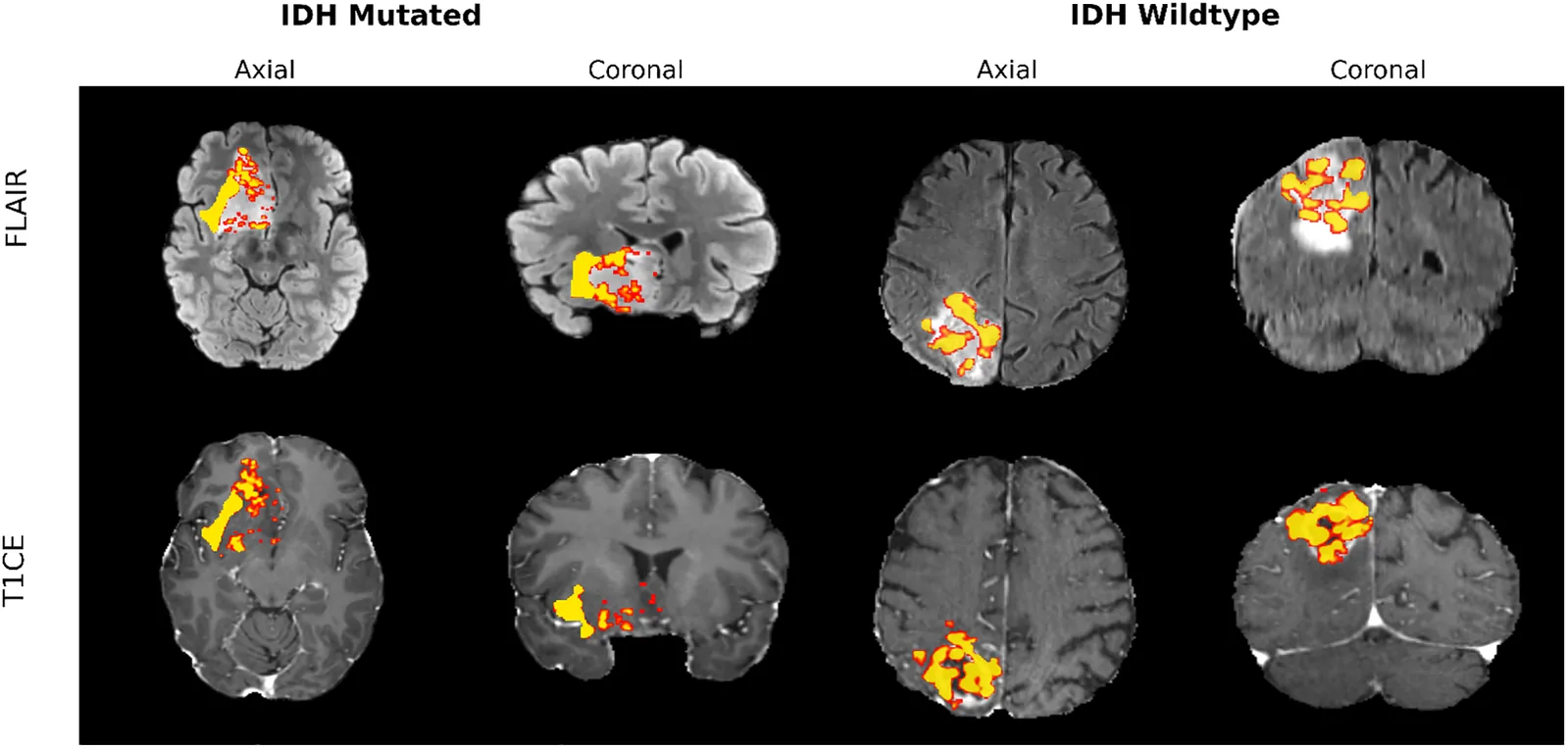
Representative examples of Integrated Gradient saliency maps show regions contributing to IDH mutation classification. Two patients are shown, stratified by IDH mutation status. Axial and coronal slices of FLAIR (top) and contrast-enhanced T1-weighted (bottom) MR images are displayed for each case. Warm colors (yellow–red overlays) indicate voxels with higher attribution to the model’s prediction.

IDH-mutant gliomas generally exhibit less contrast enhancement, whereas IDH-wildtype gliomas are more likely to demonstrate pronounced and heterogeneous enhancement, reflecting their more aggressive biology (19). This pattern appeared in the IG maps (**Figure 6**), showing that for a representative IDH-wildtype case, attributions were concentrated within enhancing regions on T1CE. In an IDH-mutant case, contributions focused on peritumoral FLAIR hyperintensity. These representative IG maps provide spatial examples consistent with the cohort-level Shapley analysis, in which FLAIR accounted for most of the predictive weight.

Shapley analysis across the test dataset found that FLAIR accounted for 64% of the predictive weight, and T1CE for 36%. This cohort-level modality distribution highlights the importance of FLAIR signal in distinguishing IDH-mutant gliomas. Conversely, contrast enhancement on T1CE provided additional information more closely linked to IDH-wildtype tumors.

## Discussion

We developed and validated an explainable 3D ensemble DL framework for non-invasive prediction of IDH mutation and MGMT methylation in gliomas. The models showed strong internal performance for IDH (validation AUC 0.94 [95% CI, 0.81-1.00]; testing AUC 0.94 [95% CI, 0.84-1.00]) and moderate external performance (AUC 0.77 [95% CI, 0.71-0.82]), while MGMT prediction was inconclusive externally (AUC 0.57 [95% CI, 0.52-0.63]). This is among the first studies to address both biomarkers using a 3D CNN ensemble and to provide explainability. Saliency analysis with IG and Shapley values highlighted sequence- and region-specific contributions, emphasizing the model’s interpretability.

Our results align with previous CNN and radiomics studies. Choi et al. reported an AUC of 0.95 for IDH status prediction using an attention-based CNN trained on dynamic susceptibility contrast perfusion MRI, but their model was developed from data from a single institution (20). Additionally, Choi et al. later evaluated a hybrid CNN-radiomics pipeline across different institutions, where performance dropped on external validation in the TCIA dataset to an AUC of 0.86 from 0.96 in the internal dataset, similar to the decline observed in our study (21). A recent meta-analysis of 39 studies found a pooled sensitivity of 0.86 and an internal validation AUC of 0.93 for IDH prediction using deep learning. Our ensemble model performed similarly, with a sensitivity of 0.90 in external validation and an internal AUC of 0.94 (22). Wu et al. developed a multi-task CNN with external AUCs from 0.71 to 0.90 for IDH predictions across three cohorts. Byeon et al. reported IDH AUCs of 0.915 on TCGA and 0.981 on UCSF, supported by visual evidence transformers (23, 24). Koska et al. showed an AUC of 0.90 for MGMT prediction using an explainable 3D CNN model with Grad-CAM visualization (15). A review of 31 studies on DL- and radiomics-based prediction of MGMT reported a pooled AUC of 0.86, which decreased to 0.69 in external validation, highlighting challenges in standardization. Few studies included external validation or explainability, underscoring the need for explainable AI to boost clinician trust (25). Saeed et al. (2023) evaluated ResNet, DenseNet, EfficientNet, ViT, and Swin Transformer architectures across two large MGMT datasets and reported mean AUCs consistently between 0.54 and 0.63 regardless of model complexity or modality combination, concluding that MRI alone may be insufficient to reliably predict MGMT status (26). Our external MGMT AUC of 0.57 falls within this near-chance range, reinforcing that this arm of our model is best interpreted as negative or inconclusive rather than clinically actionable, in clear contrast to our stronger and more consistent IDH performance. Our study nonetheless advances the field by simultaneously modeling both biomarkers using volumetric deep learning, rigorous external validation, and voxel-level interpretability. Predicting dual biomarkers is clinically significant. IDH mutation status aids in prognosis and in tailored treatments such as vorasidenib, which may improve progression-free survival and delay future interventions (27). MGMT promoter methylation remains the best indicator of benefit from temozolomide, especially in IDH-wildtype glioblastoma. It guides treatment and is associated with longer overall and progression-free survival, particularly with temozolomide (3, 27). Current biomarker assessment mainly depends on surgical sampling or biopsy, which can be limited by tumor location, spatial heterogeneity of the underlying mutation, and sampling error. MGMT methylation status in particular lacks a universal assay standard — pyrosequencing, methylation-specific PCR, and MS-MLPA apply different thresholds, and borderline-methylated cases (5–15%) show poor inter-assay reproducibility, independent of any imaging-based approach (14, 28). An imaging-based tool could supplement histopathology with non-invasive, whole-tumor evaluation before treatment. Beyond imaging-based prediction, IDH and MGMT status interact with other established prognostic factors in glioma. Histopathological grading retains independent prognostic value in IDH-mutant grade II/III gliomas, even after adjusting for 1p/19q codeletion status, indicating that molecular classification alone does not fully capture prognostic heterogeneity (29). Similarly, clinical variables such as age, sex, Karnofsky Performance Status, and extent of resection further modulate outcomes in glioblastoma alongside MGMT methylation status (30). Our non-invasive imaging-based profiling of IDH and MGMT should therefore be viewed as complementary to, rather than a replacement for, this broader landscape of histopathological and clinical prognostic factors.

The study has limitations. The decline in external performance, particularly for MGMT prediction, is most likely driven by the treatment-related domain shift inherent to the external cohort, which comprised exclusively post-treatment examinations, whereas our models were trained and validated on preoperative imaging alone. Differences in scanner hardware and acquisition protocol across institutions may have contributed additionally. Similar performance drops are reported in other multi-cohort CNN studies, underscoring the need for harmonization methods such as federated learning. Additionally, including biomarkers such as 1p/19q and ATRX, alongside IDH and MGMT, could better align with the WHO classification standards. Because IDH and MGMT labels were not jointly available for all cases, biomarker-specific cohorts may differ in case composition, introducing potential selection bias. Therefore, direct comparison between IDH and MGMT model performance should be interpreted cautiously. Another limitation is the dependence on tumor segmentation. Masks were used for cropping and image masking in both tasks, and MGMT also used the mask as an input channel. This may allow the model to learn tumor size or shape rather than molecular phenotype alone. Prospective use would require manual or automated segmentation, and future work should test models without masks and with fully automated segmentations.

A further limitation concerns the composition of the external cohort. MU-Glioma-Post comprises exclusively post-treatment examinations acquired after surgical resection and, in most cases, chemoradiation, and contains no treatment-naive baseline imaging (31) whereas the models were trained, validated, and tested entirely on preoperative studies. Treatment effects—resection cavities, blood products, post-surgical enhancement, radiation-induced FLAIR hyperintensity, and pseudoprogression—alter the same T1CE and FLAIR characteristics on which the models rely, and the overlap between treatment-related change and residual or progressive tumor on conventional MRI is well recognized (31). Some degradation in external performance is therefore expected on imaging grounds alone, and this effect is confounded with the scanner, protocol, and population shifts noted above. This does not, however, invalidate the intended preoperative application. The molecular labels themselves remain biologically anchored: IDH mutation is a very early clonal event in gliomagenesis (32) and driver alterations detectable at diagnosis are largely retained at recurrence with little evidence of recurrence-specific driver gene alteration (33). An IDH-mutant tumor therefore remains IDH-mutant on post-treatment imaging, and external evaluation under these conditions functions as a conservative stress test; the external IDH AUC of 0.77 (95% CI, 0.71–0.82) is best read as a lower bound on preoperative performance rather than as an estimate of it. The situation differs for MGMT, where promoter methylation status is less stable over the disease course: in one surgical series, MGMT methylation status determined by methylation-specific PCR changed between the initial and recurrent specimen in 38.5% of patients who had received therapeutic intervention (n = 13), compared with 12.5% of untreated recurrences (n = 8) (34) However, a substantially larger genome-wide methylation analysis of 418 matched primary-recurrent samples found the MGMT methylome to be highly stable at progression, with promoter methylation status changing in only 7% of cases (35) Given this, the post-treatment label mismatch is a plausible but not well-established contributor to the near-chance external MGMT AUC of 0.57 (95% CI, 0.52–0.63); the intrinsic limits of MRI-based MGMT prediction reported by Saeed et al. likely remain the most dominant explanation (25). Confirmation of preoperative generalizability will require an independent, treatment-naive external cohort, which we identify as the priority next step; conversely, dedicated retraining on post-treatment imaging would be required before the framework could be applied in the surveillance setting.

In conclusion, we present an explainable 3D ensemble deep learning framework capable of predicting IDH mutation status from routine MRI, with strong internal and moderate external performance. The same framework achieved strong internal performance for MGMT methylation status but failed to generalize externally, highlighting a key limitation of current radiogenomic AI and an open target for future work. Future studies should pursue large-scale prospective validation, harmonized multi-institutional collaborations, and integration of additional imaging modalities to improve generalizability — particularly for non-invasive MGMT prediction — and support clinical adoption.

## Data Availability

The datasets presented in this study can be found in online repositories. The data analyzed in this study are publicly available through The Cancer Imaging Archive (TCIA) repository (https://www.cancerimagingarchive.net). The specific datasets used are: A) UCSF Preoperative Diffuse Glioma MRI (UCSF-PDGM), https://www.cancerimagingarchive.net/collection/ucsf-pdgm/; B)UPENN-GBM, https://www.cancerimagingarchive.net/collection/upenn-gbm/; C) https://www.cancerimagingarchive.net/collection/mu-glioma-post/. All datasets are fully de-identified and accessible without restriction.
