# Supplementary material for: Externally validated explainable 3D CNN ensemble model for non-invasive prediction of IDH and MGMT status in gliomas
Source: Front Oncol. 2026 Aug 26;16:1790776. doi: 10.3389/fonc.2026.1790776 (PMC13553224; doi:10.3389/fonc.2026.1790776)
Supplement: Supplementary file 1 [file DataSheet1.docx]

**Scanners and Acquisition Parameters**
Regarding the UPENN-GBM dataset, it includes images from 1.5T and 3T Siemens and GE systems, mainly Siemens. Siemens scanners include TrioTim, Verio, Espree, Avanto, Aera, SymphonyTim, Skyra/Skyra_fit, and MAGNETOM Vida; GE scanners include Discovery MR750w, Optima MR450w, and Genesis Signa. The imaging protocol involves pre- and post-contrast 3D T1 IR-prepped FSPGR (T1 BRAVO), FLAIR, DTI, and DSC sequences.

For the UCSF-PDGM dataset, preoperative MRI was obtained using a 3.0T GE Discovery 750. The protocol includes 3D T2, T2/FLAIR, SWI, DWI, pre- and post-contrast T1, 3D ASL, DSC, and 2D 55-direction HARDI. Contrast agents used were gadobutrol at 0.1 mL/kg and gadoterate at 0.2 mL/kg.

**Detailed preprocessing steps**

The four preprocessing steps described in the main text are analyzed in further detail here. Having determined which scans would comprise the unified dataset, the samples were pre-processed in four steps. Firstly, since all UCSF and UPENN volumes were initially of shape 240 × 240 × 155, and a large proportion of them consisted of background voxels, cropping was deemed necessary for reducing RAM/GPU memory demands and noise during training, using the smallest bounding box that fit all tumors. Tumor shape for each volume was known from the segmentation volumes, and voxel volume was already consistent within the original datasets, namely 1mm × 1mm × 1mm = 1mm^3^. The scan shape was reduced to 156 × 173 × 138, and tumors were centred with relation to the final bounding box. The memory reduction within that first preprocessing step was significant; from 240 × 240 × 155 ($\approx$71.4 MB for each T1CE, FLAIR, and segmentation volume due to NIFTI float64 precision) to 156 × 173 × 138 ($\approx$29.8 MB).

Secondly, the three volumes per sample were concatenated to 156 × 173 × 138 × 3, where the channels are segmentation, T1CE, and FLAIR for the MGMT case, and to 80 × 110 × 80 × 2, where the channels are T1CE, and FLAIR for the IDH case. At each sample, the T1CE and FLAIR channels were multiplied voxel-wise with the segmentation volume to remove the remaining brain tissue, replacing the corresponding voxel values with zero. This step was included, aiming to help the models focus better on the tumor region, where most of the meaningful information is usually inferred.

The third preprocessing step includes normalization and percentile-based, outlying-voxel exclusion. Specifically, for each sample, each of the T1CE and FLAIR channels is separately treated by clipping within the 1^st^ and 99^th^ percentile range. Then, the mean and standard deviation of each clipped distribution are evaluated, excluding background voxels so that the values are not biased due to the large number of zero-valued background voxels, and used to z-score the entire channel, including background voxels (distribution centred at zero and scaled to unit variance). Furthermore, the resulting distribution is again clipped for increased robustness, within the 0.5^th^ and 99.5^th^ percentiles.

The fourth and final step consists of augmentation. Due to the relatively small amount of training samples (507 in total), augmentation was applied to the training set, aiming to improve model generalization and avoid overfitting to the training samples. Three types of augmentation were chosen: random flipping along any of the three anatomical axes (each with 50% probability), random in-plane rotation in the axial view by 0°, 90°, 180°, or 270°, or random intensity scaling by a factor uniformly sampled in the range [0.9, 1.1]. These augmentations introduced geometric and intensity variability consistent with acquisition differences while preserving the underlying tumor morphology. For each training subject, two augmented copies were generated in addition to the original sample, resulting in almost a three-fold expansion of the training set.

After preprocessing, each sample of shape 156 × 173 × 138 × 3 is converted to a float32 NumPy ndarray, further reducing memory requirements, from 71.4 MB per channel to around 14.2 MB per channel, corresponding to over 80% reduction.

**Dataset Composition for the MGMT and IDH Cases**

The splitting of the unified dataset to training, validation and testing subsets is shown in Table 1.

| **MGMT Status Classification** | | | | | | | | | |
| --- | --- | --- | --- | --- | --- | --- | --- | --- | --- |
|  | **UCSF** | | | **UPENN** | | | **Total** | | |
|  | **0** | **1** | **0 & 1** | **0** | **1** | **0 & 1** | **0** | **1** | **0 & 1** |
| **Training** | 98 | 177 | 275 | 154 | 78 | 232 | 252 | 255 | 507 |
| **Validation** | 6 | 11 | 17 | 10 | 4 | 14 | 16 | 15 | 31 |
| **Testing** | 9 | 11 | 20 | 5 | 4 | 9 | 14 | 15 | 29 |
| **Total** | 113 | 199 | 312 | 169 | 86 | 255 | 282 | 285 | 567 |
| **IDH Status Classification** | | | | | | | | | |
|  | **UCSF** | | | **UPENN** | | | **Total** | | |
|  | **0** | **1** | **0 & 1** | **0** | **1** | **0 & 1** | **0** | **1** | **0 & 1** |
| **Training** | 84 | 89 | 173 | 96 | 97 | 193 | 180 | 186 | 366 |
| **Validation** | 7 | 5 | 12 | 7 | 9 | 16 | 14 | 14 | 28 |
| **Testing** | 4 | 9 | 13 | 11 | 6 | 17 | 15 | 15 | 30 |
| **Total** | 95 | 103 | 198 | 114 | 112 | 226 | 209 | 215 | 424 |

Table 1: Classification sample composition used for model training, validation, and testing. Counts reflect preprocessed model input samples; therefore, UPENN MGMT counts may differ from the unique-case population counts in the main manuscript table.

**Hyperparameter Optimization** **for Convolutional MGMT status Classifiers**

For the individual convolutional models in the MGMT case, model architecture as well as training-specific hyperparameters were optimized using Optuna’s Tree-structured Parzen Estimator (TPE) with 500 trials, which models the posterior over hyperparameters by fitting separate kernel density estimates (KDEs) to the favourable and unfavourable trial sets, defined by a performance quantile.

The hyperparameters selected for optimization can be seen in Table 2, and the values yielded by Optuna for each of the four selected models can be seen in Table 3. All the models have the Flatten type of head, which indicates that although parameter-heavy, it was necessary to retain the entire layout of the volumes, whereas GAP failed to provide comparable results. Moreover, most of the models have ReLU activation, and a relatively large dropout rate of around 0.41, revealing that there as a tendency for overfitting, which prompted the optimizer to counterbalance it with high dropout rate. This tendency is consistent with the use of Flatten which tends to promote overfitting, along with the relatively large number of convolutional blocks and their increased dimensions. Overall, the selected architectures point to a need for detailed but parameter-heavy layout preservation, and significant countermeasures to avoid overfitting using increased dropout rate, thereby ultimately preserving meaningful information.

| **Hyperparameter** | **Search interval type** | **Search interval range** |
| --- | --- | --- |
| Learning rate | Continuous (log-scale) | [10^-6^, 10^-3^] |
| Dropout | Continuous | [0.0, 0.6] |
| Activation | Categorical | {ReLU, SeLU, Swish} |
| Head | Categorical | {Flatten, Global Average Pooling} |
| Number of convolution blocks | Integer | [1, 7] |
| Filters per block | List of integers (log-scale) | [4, 128] |
| Kernel size per block | List of integers | [3, 5] |
| Pool size per block | List of integers | [1, 2] |
| Number of Dense layers | Integer | [1, 3] |
| Neurons per Dense layers | List of integers (log-scale) | [8, 256] |

Table 2: Model hyperparameter optimization settings for the MGMT convolutional classifiers.

| **Hyperparameter** | **Model 1** | **Model 2** | **Model 3** | **Model 4** |
| --- | --- | --- | --- | --- |
| Learning rate | 1.29×10^-4^ | 5.07×10^-6^ | 1.17×10^-5^ | 2.11×10^-5^ |
| Dropout | 0.0811 | 0.4123 | 0.4109 | 0.4389 |
| Activation | Swish | ReLU | ReLU | ReLU |
| Head | Flatten | Flatten | Flatten | Flatten |
| Number of conv. blocks | 6 | 7 | 7 | 7 |
| Filters per block | 64, 8, 4, 8, 128, 32 | 4, 128, 16, 64, 32, 32, 16 | 4, 128, 16, 64, 64, 8, 16 | 4, 128, 16, 64, 64, 8, 16 |
| Kernel size per block | 3, 5, 3, 5, 5, 3 | 3, 5, 5, 5, 3, 5, 5 | 3, 5, 5, 5, 3, 5, 5 | 3, 5, 5, 5, 3, 5, 5 |
| Pool size per block | 1, 2, 1, 2, 1, 1 | 1, 2, 2, 2, 2, 2, 2 | 1, 2, 2, 2, 2, 2, 2 | 1, 2, 2, 2, 2, 2, 2 |
| Number of Dense layers | 1 | 1 | 1 | 1 |
| Neurons per Dense layers | 16 | 32 | 32 | 32 |

Table 3: Hyperparameters for the selected convolutional architectures of the MGMT case.

**Hyperparameter Optimization for MGMT Ensemble Metaclassifier**

XGBClassifier hyperparameters (see Table 4) were optimized using Optuna (TPE) over 300 trials with 15 parallel jobs for efficiency, aiming to maximize Matthews Correlation Coefficient (MCC) defined in Eq. 1, after threshold optimization. The ensemble threshold was selected by maximizing MCC on validation set probabilities during ensemble training with training set probabilities, and was used to evaluate model performance on the test set and the external validation set. The final metaclassifier architecture was evaluated on the testing set. Notably, MCC was chosen as it penalizes both types of error (FP, FN) fairly, and provides a balanced, single-number summary that incorporates all four entries of the confusion matrix (TP, TN, FP, FN), yielding a symmetric correlation measure [-1,1] that remains informative under severe class imbalance, unlike accuracy or F1, which can be biased by majority-class prevalence or ignore true negatives. The values yielded by Optuna can be seen in Table 5.

| **Hyperparameter** | **Search interval type** | **Search interval range** |
| --- | --- | --- |
| Number of estimators | Integer | [200, 1500] |
| Maximum tree depth | Integer | [2, 10] |
| Learning rate | Continuous (log-scale) | [0.001, 0.3] |
| Row sampling per tree | Continuous | [0.4, 1.0] |
| Column sampling per tree | Continuous | [0.4, 1.0] |
| Minimum loss reduction (gamma) | Continuous | [0.0, 10.0] |
| Min sum Hessian in child node | Integer | [1, 20] |
| L1 regularization | Continuous | [0.0, 10.0] |
| L2 regularization | Continuous | [0.0, 10.0] |
| Class imbalance weight | Continuous | [0.3, 5.0] |
| Max step for leaf weight | Integer | [0, 5] |

Table 4: MGMT Metaclassifier hyperparameter optimization settings.

$$MCC=\frac{TP\cdot TN-FP\cdot FN}{\sqrt{\left( TP+FP \right)\left( TP+FN \right)\left( TN+FP \right)\left( TN+FN \right)}}$$

Eq. 1

| **Hyperparameter** | **Value** |
| --- | --- |
| Number of estimators | 456 |
| Maximum tree depth | 2 |
| Learning rate | 0.1339 |
| Row sampling per tree | 0.4387 |
| Column sampling per tree | 0.9108 |
| Minimum loss reduction (gamma) | 1.3678 |
| Min sum Hessian in child node | 19 |
| L1 regularization | 4.2312 |
| L2 regularization | 4.1581 |
| Class imbalance weight | 3.3187 |
| Max step for leaf weight | 3 |

Table 5: Hyperparameters for the selected XGBoost metaclassifier architecture of the MGMT case.

**Explainability**

Two correctly classified samples were selected, one from each class. Integrated gradients were computed for each sample ($\boldsymbol{x}$) with respect to an all-zero baseline ($\boldsymbol{x}_{bl}$), a natural absence-of-signal reference. M=128 steps across the straight line from baseline to input were utilized, each with a different, equally spaced value for α, as shown in Eq. 2. Additionally, N = 8 SmoothGrad-IG repeats were performed by adding small Gaussian noise to the input from a distribution with mean 0.0 and standard deviation 5% of input standard deviation, before recomputing IG and averaging, which reduces high-frequency noise. The formula for Integrated Gradients is shown in Eq. 3, where $F\left( \cdot\right)$ signifies the model operator, thus $F\left( \boldsymbol{x} \right)$ is the convolutional model output (class-1 probability) for input $\boldsymbol{x}$, and $\odot$ signifies voxel-wise multiplication, not to be confused with matrix multiplication. The integral is approximated with a Riemann sum as in Eq. 4, whereas the gradient of $F$ with respect to $\boldsymbol{x}$ evaluated at $\boldsymbol{x}_{\alpha}$, namely $\boldsymbol{\nabla}_{\boldsymbol{x}}F\left( \boldsymbol{x}_{\alpha} \right)$, is computed using TensorFlow automatic differentiation.

$$\boldsymbol{x}_{\alpha}=\boldsymbol{x}_{bl}+\alpha\left( \boldsymbol{x}-\boldsymbol{x}_{bl} \right)$$

Eq. 2

$$IG\left( \boldsymbol{x} \right)=\left( \boldsymbol{x-}\boldsymbol{x}_{bl} \right)\odot\int_{0}^{1} \boldsymbol{\nabla}_{\boldsymbol{x}}F\left( \boldsymbol{x}_{bl}+\alpha\left( \boldsymbol{x}-\boldsymbol{x}_{bl} \right) \right)d\alpha$$

Eq. 3

$$IG\left( \boldsymbol{x} \right)\approx\left( \boldsymbol{x-}\boldsymbol{x}_{bl} \right)\odot\frac{1}{M}\sum_{k=1}^{M} \boldsymbol{\nabla}_{\boldsymbol{x}}F\left( \boldsymbol{x}_{bl}+\frac{k}{M}\left( \boldsymbol{x}-\boldsymbol{x}_{bl} \right) \right)$$

Eq. 4

Additionally, each MRI modality’s contribution to the classification probability was assessed with exact Shapley values. To compute them for each test set sample, the model received the input volume each time, removing one of the channels by setting all its voxel values to zero, as a baseline matching the background. After determining the exact Shapley values, channel-wise contributions were reported as percentages per sample and averaged across the test set to capture the underlying trend.

**Hyperparameter Optimization for Convolutional IDH Status Classifiers**

Model architecture as well as training-specific hyperparameters were optimized using Optuna’s Tree-structured Parzen Estimator (TPE) with 500 trials. The hyperparameters selected for optimization are displayed on Table 6, and the values yielded by Optuna can be seen in Table 7. Several common trends can be found among the models. For instance, L2 kernel regularization is consistently low but nonzero, and dropout rate averages around 0.15, a reasonably moderate value which allows Dense layers to capture information without overfitting. Similarly, spatial dropout rate averages close to 0.13, providing a comparable regularization effect to the convolutional layers. On most models, initial convolutional blocks tend to contain Conv3D layers with more filters. This indicates that the models needed a stronger feature extraction capacity before downsampling, as finer details in the tumor texture are still distinguishable. After downsampling, fewer filters sufficed to capture coarse trends in tumor morphology. It should be noted that only the 1^st^ convolutional block’s pooling size is configurable, and the rest are equal to 2.

| Hyperparameter | Search interval type | Search interval range |
| --- | --- | --- |
| Learning rate | Continuous (log-scale) | [10^-5^, 10^-4^] |
| L2 kernel regularization | Continuous (log-scale) | [10^-6^, 10^-4^] |
| Dropout rate | Continuous | [0.0, 0.35] |
| Spatial Dropout rate | Continuous | [0.0, 0.25] |
| Activation | Categorical | {ReLU, GELU} |
| Downsampling mode | Categorical | {strided Conv3D, MaxPool3D} |
| Number of conv. blocks | Integer | [3, 6] |
| Filters per block | List of integers (log-scale) | [0, 256] |
| Pool size on 1st block | Integer | [1, 2] |
| Number of Dense layers | Integer | [0, 2] |
| Neurons per Dense layer | List of integers (log-scale) | [0, 8] |

Table 6: Model hyperparameter optimization settings for the IDH case.

| **Hyperparameter** | **Model 1** | **Model 2** | **Model 3** | **Model 4** |
| --- | --- | --- | --- | --- |
| Learning rate | 5.35×10^-5^ | 8.62×10^-5^ | 8.28×10^-5^ | 6.63×10^-5^ |
| L2 kernel regularization | 1.72×10^-6^ | 1.44×10^-6^ | 4.06×10^-6^ | 1.93×10^-6^ |
| Dropout rate | 0.24 | 0.13 | 0.09 | 0.12 |
| Spatial Dropout rate | 0.17 | 0.09 | 0.07 | 0.19 |
| Activation | GELU | ReLU | ReLU | ReLU |
| Number of conv. blocks | 4 | 5 | 4 | 4 |
| Filters per block | 32, 16, 16, 1 | 64, 64, 8, 1, 4 | 64, 128, 16, 2 | 8, 1, 128, 2 |
| Strided block index | 0 | 1 | 1 | 0 |
| Pool size on 1st block | 1 | 1 | 1 | 1 |
| Number of Dense layers | 1 | 1 | 1 | 1 |
| Neurons per Dense layer | 1 | 1 | 1 | 1 |

Table 7: Hyperparameters for the best four of the selected convolutional architectures of the IDH case.

**IDH Ensemble Metaclassifier**

The ensemble metaclassifier for the IDH case received as input the convolutional model probabilities, outputting an improved value, equal to the weighted sum of the input probabilities. The weights added up to 1.0, and were selected by solving a constrained optimization problem, defined in Eq. 5. Specifically, the objective of the problem is to compute the weights vector $\boldsymbol{w}$ which minimizes $\left\| y-\boldsymbol{P}\cdot\boldsymbol{w} \right\|^{2}$, where $y\in\{0, 1\}$ is the corresponding true label and $\boldsymbol{P}$ is the individual model probabilities vector. Minimizing this metric optimally leads to $y=\boldsymbol{P}\cdot\boldsymbol{w}$ the best-case scenario of the metaclassifier reaching 100% certainty of correct classification.

| $\min\left\Vert y-\boldsymbol{P}\cdot\boldsymbol{w} \right\Vert^{2} while w_{j}\geq0 and\sum_{j} w_{j}=1$ | Eq. 5 |
| --- | --- |
| The ensemble threshold was selected by maximizing Youden’s J, which is equivalent to maximizing $\left( TP-FP \right)$, during ensemble training and was used to evaluate model performance on the test set and the external validation set. |  |

**Precision-Recall and Calibration Analyses**

Supplementary Figure 1: Precision-recall curves for the MGMT and IDH ensemble models across validation, testing, and external validation sets. Dashed lines indicate class-1 prevalence.

Supplementary Figure 2: Calibration curves for the MGMT and IDH ensemble models across validation, testing, and external validation sets. The diagonal line indicates ideal calibration.

| **Biomarker** | **Set** | **n** | **Class 0** | **Class 1** | **Class 1 prevalence** | **Average precision** | **Brier score** |
| --- | --- | --- | --- | --- | --- | --- | --- |
| MGMT | Validation | 31 | 16 | 15 | 0.484 | 0.769 | 0.264 |
| MGMT | Testing | 29 | 14 | 15 | 0.517 | 0.655 | 0.291 |
| MGMT | External | 482 | 295 | 187 | 0.388 | 0.450 | 0.403 |
| IDH | Validation | 28 | 14 | 14 | 0.500 | 0.908 | 0.191 |
| IDH | Testing | 30 | 15 | 15 | 0.500 | 0.944 | 0.174 |
| IDH | External | 558 | 462 | 96 | 0.172 | 0.418 | 0.235 |

Supplementary Table 8: Average precision and Brier scores for ensemble probability estimates across validation, testing, and external validation sets.
